# On the feasibility of the computational modelling of the endoluminal vacuum-assisted closure of an oesophageal anastomotic leakage

**DOI:** 10.1098/rsos.171289

**Published:** 2018-02-07

**Authors:** Ester Comellas, Facundo J. Bellomo, Iván Rosales, Luis F. del Castillo, Ricardo Sánchez, Pau Turon, Sergio Oller

**Affiliations:** 1 International Center for Numerical Methods in Engineering (CIMNE), Campus Nord, Building C1, c/Gran Capità s/n, 08034 Barcelona, Spain; 2 INIQUI (CONICET), Faculty of Engineering, National University of Salta, Av. Bolivia 5150, 4400 Salta, Argentina; 3 B. Braun Surgical, S.A., Carretera Terrassa, 121, 08191 Rubí, Barcelona, Spain; 4 Department of Civil and Environmental Engineering, ETSECCPB, Universitat Politècnica de Catalunya, Barcelona Tech (UPC), Campus Nord, Building C1, c/Jordi Girona 1-3, 08034 Barcelona, Spain

**Keywords:** endoluminal vacuum-assisted closure, healing, growth, finite-element analysis, constitutive modelling

## Abstract

Endoluminal vacuum-assisted closure (E-VAC) is a promising therapy to treat anastomotic leakages of the oesophagus and bowel which are associated with high morbidity and mortality rates. An open-pore polyurethane foam is introduced into the leakage cavity and connected to a device that applies a suction pressure to accelerate the closure of the defect. Computational analysis of this healing process can advance our understanding of the biomechanical mechanisms at play. To this aim, we use a dual-stage finite-element analysis in which (i) the structural problem addresses the cavity reduction caused by the suction and (ii) a new constitutive formulation models tissue healing via permanent deformations coupled to a stiffness increase. The numerical implementation in an in-house code is described and a qualitative example illustrates the basic characteristics of the model. The computational model successfully reproduces the generic closure of an anastomotic leakage cavity, supporting the hypothesis that suction pressure promotes healing by means of the aforementioned mechanisms. However, the current framework needs to be enriched with empirical data to help advance device designs and treatment guidelines. Nonetheless, this conceptual study confirms that computational analysis can reproduce E-VAC of anastomotic leakages and establishes the bases for better understanding the mechanobiology of anastomotic defect healing.

## 1. Background

Anastomotic leakages are associated with high morbidity and mortality rates in both oesophageal and colorectal anastomoses [1,2]. Following oesophageal anastomosis, patients that develop leaks have a threefold death risk [2] than those who do not, with mortality reaching up to 60% in the former group [3,4]. The prevalence of anastomotic leakages varies according to anatomical site, but values as high as 19% have been recorded in colorectal anastomoses [5]. Studies in the past years have identified the main factors associated with this detrimental post-surgical complication, contributing to improve the clinical outcome through advances in patient selection and adequate pre- and peri-operative procedures (see [5–8] and references therein). Among these, the most promising therapeutic agents for its prevention are the promotion of angiogenesis and improvement of tissue perfusion [9–11], together with the inhibition of the degradation of collagen and acceleration of granulation tissue deposition and epithelialization [10,12,13]. Yet, the incidence of anastomotic leaks has not decreased significantly [14], which confirms that the fundamental mechanisms at play in their formation remain unclear [15].

Failure of anastomotic healing is a life-threatening condition that must be treated immediately. Traditionally, it required either reoperation or conservative treatment. In recent years, the development of endoscopic techniques have brought new and less invasive surgical procedures to the clinical practice, including the use of stents and clips, application of plugs together with fibrin glue, and endoscopic insertion of drainages [4,16–18].

Endoluminal vacuum-assisted closure (E-VAC) is a promising therapy for the treatment of anastomotic leakage in the oesophagus and bowel. An open-pored polyurethane foam attached to a portable vacuum pump via a flexible tube is introduced into the leakage cavity formed at the anastomotic site. Controlled suction is applied to drain the secretions accumulated in the cavity, thereby decreasing bacterial contamination and local oedema. At the same time, the vacuum therapy promotes the formation of granulation tissue, accelerating the healing of the defect. Note that this process does not entail healing of the tissue as understood in the classical sense [19,20] but, rather, seeks the reduction of the cavity’s volume.

Vacuum-assisted closure is widely accepted by clinicians as an efficient method to promote healing in open and difficult wounds [21,22], and has been successfully used over the past decades [23–26]. More recently, the technique has been adapted to endoscopic application to effectively treat colorectal [27–30] and oesophageal leaks [16,31–36].

There are numerous clinical and preclinical studies trying to elucidate the mechanisms of action of VAC therapy [23,24,37–40]. Three primary mechanisms of action have been identified in open wound VAC, which are also applicable to E-VAC: (i) macrodeformation or wound shrinkage; (ii) microdeformation at the foam–wound surface interface; and (iii) fluid removal [22]. The suction pressure leads to a significant decrease in the wound surface area (macrodeformation). The compression induced by this deformation increases the extracellular pressure in the tissue underlying the wound bed in both *in vivo* and *in vitro* studies. The suction is applied through the open-cell foam, creating an undulation of the wound surface due to the porosity of the material (microdeformation). This induces microscopic strain in the tissue, which is thought to contribute to cellular proliferation and angiogenesis, which promote formation of granulation tissue. Finally, the application of vacuum helps to remove the excess extracellular fluid, reducing the compression of the microvasculature and allowing for increased irrigation to the area.

Despite the promising results of E-VAC therapy in anastomotic leakages, there is an absence of exhaustive large-scale comparative studies, and the effects of the treatment variables (e.g. foam pore size or pressure range) have not been quantified in detail. Increased knowledge of the specific biomechanical mechanisms driving this therapy could lead to improvements in the device designs and help to provide better guidelines for healthcare professionals.

Finite-element (FE) modelling is extensively used in biomedical engineering in areas ranging from prosthesis and surgical device design to patient-specific biomechanical studies. Yet, there are only a few FE analyses studying the effects of VAC therapy [39,41–43]. These compute the stresses or strains on the wounded tissue induced by the application of VAC therapy, backing up the notion that mechanical forces promote wound healing. To our knowledge, no attempt has been made to study how E-VAC promotes the healing of an anastomotic cavity using computational tools. Such an FE analysis may shed light on the mechanisms of action in this healing process, contributing to advancing our knowledge of this promising therapy.

The aim of this study is to study the feasibility of numerically reproducing the E-VAC of an anastomotic leakage by means of the finite-element method (FEM). The ultimate goal is to provide tools to improve our understanding of the technique and mechanisms at play in this healing process. We take as reference the Eso-sponge^®^ device (B. Braun Surgical, S.A., Rubí, Spain) used to treat oesophageal leakages, but results can be easily extrapolated to similar devices used to treat this and other areas of the gastrointestinal tract such as the bowel. We propose differentiating between the *purely mechanical or structural* response of the tissue to the application of vacuum and the *regeneration or healing* part of the process. The latter is obviously influenced by the former, but the time scales on which these two processes act are sufficiently distinct to be able to compute them separately and, then, account for how they influence each other. The *dual-stage analysis* developed is described in §2. The constitutive model defined to reproduce the tissue behaviour as well as the overall numerical framework and its implementation are presented in §3. A computational example of a generic E-VAC of an anastomotic leakage is given in §4. The strengths and weaknesses of the computational model are discussed in §5 and the conclusion of this conceptual study is summarized in §6.

## 2. Computational approach: dual-stage analysis

**Figure 1.**
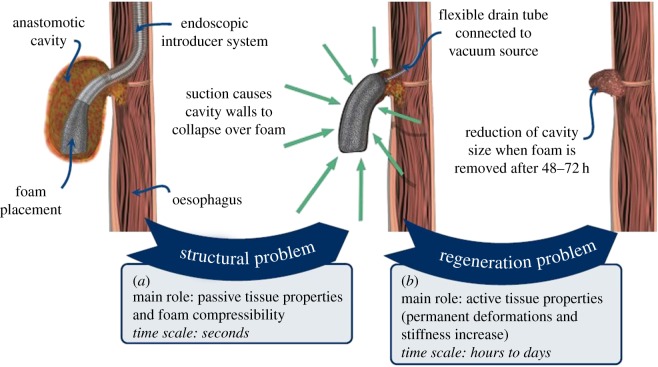
Dual-stage analysis for the computational study of the E-VAC of an oesophageal anastomosis. (*a*) The structural problem addresses the reduction of the cavity due to the application of suction pressure (centre) through the endoscopically inserted foam (left), while (*b*) the regeneration problem focuses on the healing or permanent reduction in cavity size over time (right). Images reproduced with permission from B. Braun Surgical S.A.

Following the identification of the anastomotic defect via diagnostic examination, the foam is introduced inside the cavity with the aid of the endoscopic introducer system (figure 1, left). Once the foam is in place, the endoscopic introducer system is completely removed and only the foam remains inside the cavity. It is attached to a flexible drain tube that is connected to an extracorporeal vacuum source. When the pump is activated, the open-pored polyurethane material distributes the suction pressure over the whole surface of the foam, resulting in the collapse of the cavity walls over the foam surface (figure 1, centre). The pressure is typically maintained 48–72 h to promote the formation of granulation tissue. When the foam is removed, there is a visible reduction in the cavity volume, indicating successful healing of the anastomotic defect (figure 1, right). In addition, the removal of the foam contributes to the debridement of the injury site because the foam tears the external layer of tissue which has grown into the pores. The surface damage induced is thought to further stimulate the healing. The process is repeated several times until the cavity is reduced to a minimum size and the defect can be securely closed.

From a biomechanical point of view, the E-VAC healing process described above can be split into two distinct problems, characterized by different mechanisms of action and time scales (figure 1). First, the reduction of the cavity due to the application of suction will be studied as a purely structural phenomenon. The outcome of this first phase will be the starting point for the second part of the problem, namely, the healing or reduction of the cavity over time, stimulated by the suction induced in the first phase. Through this dual-stage analysis we will reproduce the effect due to a single foam application. Then, the process will be successively repeated to capture the whole healing process, comprising several foam changes.

### 2.1. First stage: structural problem

The initial reduction in cavity size due to the application of vacuum is a direct response of the tissue to a mechanical action. The time scale of this problem is in the order of seconds, hence the active response of the tissue, i.e. its healing action during this time frame, can be neglected. Only the behaviour of the foam material and the purely mechanical properties of the tissue play a significant role in the physical contraction observed.

The foam is cut to fit in the cavity. When the vacuum pump is activated, a suction pressure is applied on the cavity walls and any fluid inside the cavity is drained. The walls can be regarded as ‘stuck’ to the foam, i.e. the foam exerts a reaction on the tissue, distributing the suction pressure and preventing large buckling of the cavity walls. This is the mechanism by which the cavity is reduced. It is on this configuration that the regeneration problem acts. If the suction pressure were released immediately after activating the pump, the cavity walls would not remain in the shrunken position. This corroborates that, at this point, the healing process leading to permanent cavity reduction is not acting yet.

**Figure 2.**
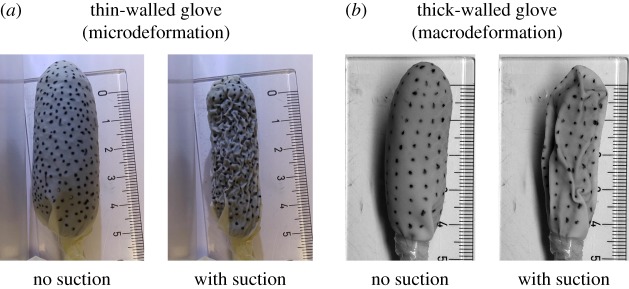
Simple suction experiments with an Eso-sponge^®^ using a flexible thin-walled cavity (medical latex glove, *a*) and a more rigid thick-walled cavity (household latex glove, *b*). The glove finger was sealed below the foam, and suction was applied through the tube.

The foam size, shape, stiffness and compressibility as well as the position and configuration of the suction tube are likely to influence the final equilibrium position. The nature of the cavity wall is also key to determining the final deformed configuration of the structural problem. Unfortunately, there is barely any literature available on the composition and characteristics of the anastomotic cavity wall tissue. To our knowledge, only Shomaf [44] attempted to describe in an exhaustive manner the histopathology of an intestinal anastomosis. The cavity wall is formed by the tissues surrounding the leakage area, covered by a layer of fascias. Generally, the leakage is contained by the adjacent structures and organs which contribute to the effective stiffness of the cavity wall. In our model, the cavity wall material will loosely represent the tissue surrounding the leakage area, and the effect of the surrounding organs on the wall’s response to suction. To better understand how the cavity wall properties influence the contraction pattern, we performed simple suction experiments with an Eso-sponge^®^ device using latex pouches as pseudo-cavities. Both wrinkling and folding were observed, which can be associated with the micro- and macrodeformation VAC mechanisms identified by Huang *et al.* [22]. These two forms of structural buckling are closely associated with the thickness and rigidity of the ‘cavity wall’ (figure 2).

### 2.2. Second stage: regeneration problem

Over the hours following the insertion of the foam, the biological processes that promote growth and remodelling of the tissue forming the surface of the cavity are activated. Here, the adaptation properties of the living injured tissue, stimulated by the pressure, play a main role in achieving the final *permanent* reduction of the cavity.

**Figure 3.**
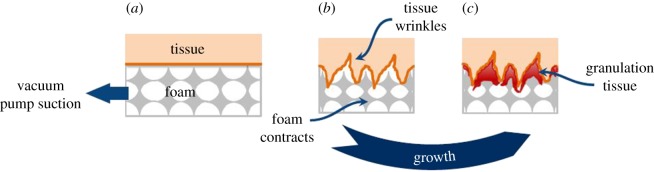
Interpretation of the permanent deformations due to microdeformations in the regeneration problem. (*a*) The suction action of the vacuum pump causes (*b*) the foam to contract, forming wrinkles in the cavity wall and promotes the (*c*) growth of granulation tissue, which fills the small wrinkles of the cavity wall and infiltrates the open pores of the foam.

The regeneration problem is assumed to act on the final deformed (non-permanent) configuration resulting from the structural problem. There is a continuous suction pressure throughout the process, keeping the cavity wall in its contracted position. After several hours, when the pressure is released to change the foam, the cavity keeps its reduced configuration. Therefore, there has been a permanent deformation in the tissue, which can be attributed to the growth of granulation tissue due to cell proliferation and angiogenesis promoted by the suction action (see [22] and references therein) (figure 3). In addition, the newly grown tissue smooths out the undulation and wrinkles of the microdeformations induced by the vacuum pressure (similar to the thin-walled glove in figure 2). Tissue that has grown into the pores is torn off with the foam when it is extracted after 48 h, suggesting that the regenerative process described above occurs in a time frame of hours, possibly between 10 and 40 h.

The suction pressure also induces a global contraction of the cavity at macroscale, which produces folds in the cavity wall. Medical images taken endoscopically by Moschler *et al.* [45] show that after placing the foam in the anastomotic cavity and applying suction over a certain period of time, the cavity takes an alveolar shape, which suggests that macroscopic folds occur due to the vacuum pressure (similar to the thick-walled glove in figure 2). In addition, tissue strands are seen across the cavity where the foam ends, which is in agreement with the idea of the cavity walls closing over the extreme of the foam, sealing the cavity. Thus, tissues forming opposing cavity walls, when adequately debrided, join together if in contact during a certain period of time, possibly in the range of hours.

In addition to the deformation phenomena observed at micro- and macroscales, the healing tissue most probably experiences the characteristic turnover of remodelling tissue, introducing stress relaxation. VAC therapy using open-cell polyurethane foam is known to accelerate the production of highly disorganized granulation tissue. By contrast, other type of foams and gauzes produce lower quantities of new tissue, albeit much more organized in nature and, thus, with higher strength and stiffness properties [40]. In such cases, there is probably a significant stiffness increase of the cavity wall tissue at the same time permanent deformations take place.

A permanent deformation is observed between foam changes (every 48–72 h), while the remodelling of the healing tissue into scar tissue, which results in a completely closed and functional cavity, takes at least several weeks [33]. Months to years are needed to achieve strength and stiffness values in the scar tissue comparable to the original uninjured tissue. This supports the idea that these are distinct processes with separate (but overlapping) time scales and possibly slightly different driving mechanisms.

We will model the reduction in the cavity size between successive E-VAC applications as a permanent deformation entailing stress relaxation, which can be phenomenologically reproduced by means of a continuum growth model [46]. The stiffness increase will be accounted for through a reverse damage model, based on the homeostatic-driven turnover remodelling (HTR) model [47]. When the tissue is growing at maximum velocity (rapid formation of disorganized granulation tissue), there will be stress relaxation but barely any stiffness increase. Once the tissue reaches a certain equilibrium and its growth rate is reduced, then the stiffness increase model will become the main driver of the healing process.

## 3. Continuum framework and numerical implementation

Biological tissues are typically modelled as quasi-incompressible hyperelastic material [48], which requires the use of a mixed displacement–pressure formulation in FE analyses [49]. On the other hand, the highly compressible hyperelastic behaviour of the foam requires the use of classic displacement-based formulation. Owing to the nature of the phenomena involved (figure 1), we use a dual-stage analysis to computationally reproduce the E-VAC of an anastomotic cavity in two separate consecutive steps. We take this approach for convenience and simplicity: our focus is on developing an adequate constitutive model, not on addressing the numerical and technical challenges derived from mixing hybrid and classic elements in a same model.

**Figure 4.**
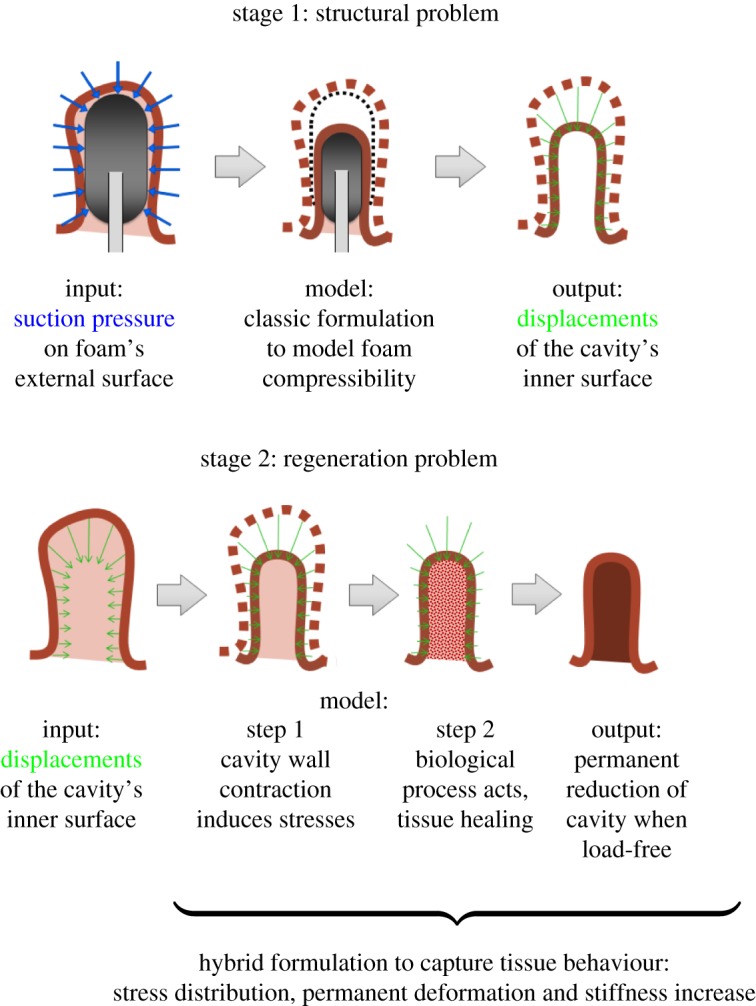
Illustration of the general concept followed in the computational modelling of a healing anastomotic cavity using the dual-stage analysis.

In the first stage, the structural problem will be modelled with the classic formulation because the priority is capturing the behaviour of the foam and its effect on the reduction of the cavity. We will obtain the displacements of the internal surface of the cavity wall due to the suction action through the foam. Stresses and strains in the tissue will not be accurate because the tissue will not behave as a quasi-incompressible material. In the second stage, these displacements will be imposed on the initial geometry of the inner surface of the cavity wall. Now the tissue will be modelled with hybrid elements and, hence, a more realistic quasi-incompressible hyperlastic material behaviour. In a first computational step, displacements will produce a stress distribution in the tissue. At this point, the regeneration process will start, and the healing of the anastomotic cavity will be driven by the stresses produced through the imposed displacement associated with the suction action of the foam. Figure 4 illustrates the computational details of the dual-stage analysis.

The new constitutive model developed to capture the behaviour of the healing tissue in the anastomotic cavity is described below. The fundamental biological aspects are not well known at present, hence we tackle the material modelling from a phenomenological point of view. We reproduce the permanent deformations (volume reduction) and stiffness increase (tissue remodelling) of the healing cavity without attempting to directly associate the underlying cellular processes at play with the macroscopically observed response. In the future, as experimental and clinical researchers manage to elucidate the fundamental biological mechanisms involved in this complex process, these can be gradually incorporated into the computational model.

### 3.1. Permanent deformations

Continuum growth is generally modelled through the decomposition of the deformation gradient tensor [50],

3.1 $$\text{F}={\text{F}}^{e}\cdot {\text{F}}^{g},$$

where **F**^e^ corresponds to the elastic part of the tensor and **F**^g^ to the incompatible part. The tensor **F**^g^ includes the permanent deformations due to the growth of granulation tissue and the readjustment of the external tissues surrounding the cavity. For the sake of simplicity, we assume isotropic volumetric growth. Then, the growth tensor is

3.2 $${\text{F}}^{g}=\vartheta \;\text{I},$$

where **I** is the identity tensor and *ϑ* is often known as the growth multiplier [51]. Therein after, this internal variable will be referred to as the *remodelling permanent stretch* because here it is related to the volume change that leads to a permanent deformation of the cavity due to remodelling of the tissue. In particular, the volume change due to growth is (1−det **F**^g^)=(1−*ϑ*^3^). For *ϑ*=1 there is no growth, values above 1 indicate growth, and values between 0 and 1 represent atrophy. Note that here we understand atrophy as a volume reduction, not a loss of function. For example, *ϑ*=0.8 will correspond to a volumetric loss of (1−0.8^3^)×100=48.8%.

Following the classical continuum growth models [52], the evolution of the remodelling permanent stretch is defined as

3.3 $$\dot{\vartheta }=f(\vartheta )\phi (\sigma^{e}),$$

where the weighting function *f*(*ϑ*) imposes limits on growth to avoid an uncontrolled tumour-like behaviour and the function *ϕ*(*σ*^e^) is a mechanically driven growth criterion.

We consider the well-known growth-limiting function [53]

3.4 $$f(\vartheta )={\left[\frac{\vartheta^{\lim}-\vartheta }{\vartheta^{\lim}-1}\right]}^{\gamma },$$

whose value diminishes from 1 to 0 as the values *ϑ* become close to the limit value *ϑ*^lim^. The parameter *γ* will allow adjusting the behaviour of the function when adequate experimental data is available but, for this study, has been set to 1.

As to the definition of the mechanical criterion *ϕ*(*σ*^e^), we take as guideline what is known on the effects of the negative pressure on the healing rate of wounds. Past studies analyse the effects of the suction pressure level in VAC therapy on initial wound contraction, blood flow in the tissue, microdeformation, fluid removal and granulation tissue formation. In general, for pressure values below 40 mmHg healing behaviour is similar to the control case without VAC (zone 1). Between 40 and 80 mmHg VAC therapy provides beneficial effects, which increase with suction pressure (zone 2). Different studies provide only partial information, yet globally seem to indicate that from 80 mmHg onwards a ceiling is reached, in which the maximum benefits of VAC therapy are obtained (zone 3). For values above 200 mmHg, negative effects such as tissue abrasion and blood vessel collapse have been reported. Not much information is available on values above 125 mmHg because the range of therapeutic action is generally limited to 150 mmHg [21].

**Figure 5.**
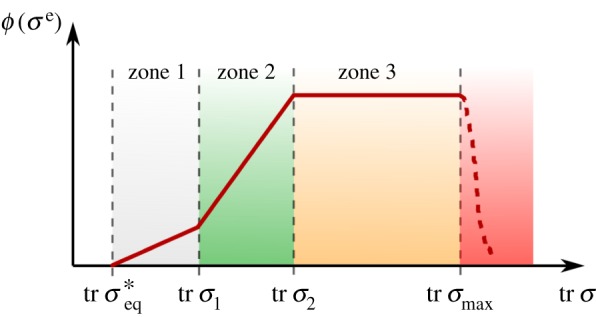
The proposed mechanical criterion *ϕ*(*σ*^e^) to control the permanent deformations is driven by the trace of the Cauchy stress tensor tr *σ*, which can be directly associated with a suction pressure value. The suction pressure will produce a compressive stress state in the tissue, hence all (positive) traces of the stress tensor shown here are in fact compressive.

Considering the above, we propose the conceptual function in figure 5, defined in terms of the trace of the Cauchy stress tensor, tr *σ*:

3.5 $$\begin{array}{rl} \phi (\sigma^{e}) & \;=k^{g}(\text{tr}\;\sigma -\text{tr}\;\sigma_{\mathrm{eq}}^{*})\;\text{if}\;\text{tr}\;\sigma_{1}\geq \text{tr}\;\sigma >\text{tr}\;\sigma_{\mathrm{eq}}^{*}\\ \phi (\sigma^{e}) & \;=k^{g}(\text{tr}\;\sigma_{1}-\text{tr}\;\sigma_{\mathrm{eq}}^{*})+k_{1}^{g}(\text{tr}\;\sigma -\text{tr}\;\sigma_{1})\;\text{if}\;\text{tr}\;\sigma_{2}\geq \text{tr}\;\sigma >\text{tr}\;\sigma_{1}\\ \;\text{and}\;\phi (\sigma^{e}) & \;=\phi_{\max}\;\text{if}\;\text{tr}\;\sigma_{\max}\geq \text{tr}\;\sigma >\text{tr}\;\sigma_{2}. \end{array}\}$$

Here, σ^*^_eq_ is the stress threshold above which the mechanical stimulus for growth begins, and tr *σ*_1_ and tr *σ*_2_ are representative values of tr *σ* in the tissue. Above tr *σ*_max_ no healing occurs.

The tr *σ* has been selected as the driving mechanism for the mechanical growth function because it depends directly on the vacuum pressure applied. When the cavity wall has contracted due to the application of pressure but the biological process has still not begun, each suction pressure value will correspond to a different stress state in the model. As tissue will be modelled with the simplest hyperelastic model available, neo-Hookean hyperelasticity, these values will be directly proportional. Therefore, performing a numerical simulation of suction for the values of interest and estimating a representative (mean) value of tr *σ* for each, allows directly relating the suction pressure to the function *ϕ*(*σ*^e^). This relation is only valid before growth begins. Once the tissue starts growing, its stress will decrease, while the suction pressure remains constant.

### 3.2. Stiffness increase

The material model for the cavity wall represents the overall stiffness behaviour of the surrounding organ tissues and the thin surface of mucosa and fascia. As the cavity closes, the tissue forming this initially thin inner layer grows and becomes stiffer. Unfortunately, we do not have enough information to distinguish between the stiffness increase due to the consolidation of the surface and the one due to the adjacent organs returning to their natural position. Hence, these two effects will be accounted for together. We use the HTR model [47], which defines the second Piola–Kirchhoff stress tensor as

3.6 $$S=S_{\mathrm{vol}}+(1-D_{\mathrm{eff}}){\tilde{S}}_{0},$$

where ***S***_*vol*_ is the volumetric part of the stress tensor and ${\tilde{S}}_{0}$ is the hyperelastic (undamaged) deviatoric part. The rate of the effective scalar damage *D*_eff_ is given by

3.7 $${\dot{D}}_{\mathrm{eff}}=\dot{D}-\dot{R},$$

where $\dot{D}$ is the rate of an explicit mechanical damage variable [54] and $\dot{R}$ is the rate of the regeneration or healing term *R*. In this study, *D*_eff_ is a representation of the stiffness increase, rather than a recovery, because the tissue at the end of the process, once the cavity closes, is not the same tissue that formed the cavity walls in the anastomotic leak. Herein after, the effective damage will be more adequately renamed as *stiffness increase index*, although the nomenclature is kept unchanged to match the HTR model definition. An initial value of *D*_eff_=0.2 in the tissue will correspond to a final stiffness increase of 20% once the E-VAC procedure is complete (and the tissue has achieved *D*_eff_=0).

The healing rate is

3.8 $$\dot{R}=\dot{\eta }\langle D_{\mathrm{eff}}-\xi \rangle ,$$

where the Macaulay brackets indicate 〈•〉=max(•,0) and *ξ*∈[0,1] defines the percentage of stiffness that is not recovered at the end of the healing process. As for the present application the effect of *ξ* is similar to establishing an initial value *D*_eff,0_ in the injured tissue, this parameter is set to zero. In the original HTR model, *D*_eff,0_ and *ξ* are conceptually different: *ξ* is the amount of damage that can never be recovered in the healed scar tissue, while an initial damage *D*_eff,0_ could be fully recovered for *ξ*=0. In the current formulation, we are interested in defining the stiffness increase of the tissue in the healed anastomotic cavity with respect to the initial tissue, so the amount of interest is the difference 〈*D*_eff,0_−*ξ*〉. As mentioned above, the materials at the beginning and end of the healing process are actually different tissues. Therefore, for a given stiffness increase, we do not have a ‘healthy’ undamaged material to use as the reference when selecting the value *ξ*. Hence, for the sake of simplicity, we choose to disregard this parameter in the present model.

**Figure 6.**
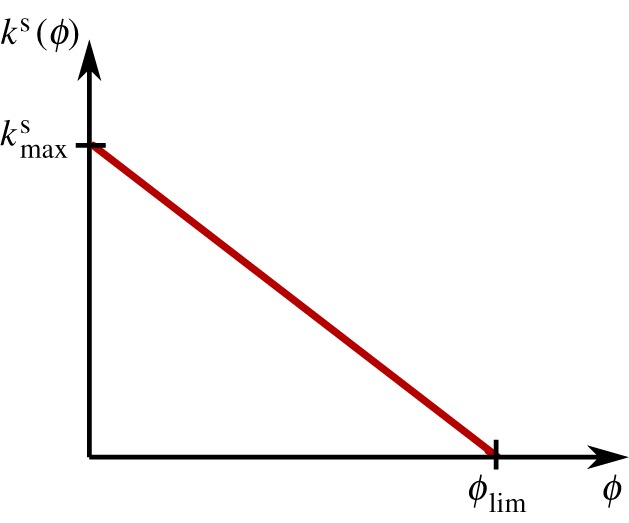
The function *k*^s^ proposed to regulate the healing speed that leads to a stiffness increase in the tissue depends on the mechanical growth rate criterion *ϕ*.

The function $\dot{\eta }$ regulates how fast the stiffness increase occurs, which is directly related to the metabolism of the system. We propose a function *k*^s^ of the mechanical growth rate criterion *ϕ*(*σ*^e^) (figure 6),

3.9 $$\dot{\eta }=k^{s}(\phi )=k_{\max}^{s}\left(1-\frac{\phi }{\phi_{\lim}}\right).$$

The proliferation and growth of granulation tissue is inversely proportional to the rate of stiffness increase up to the limit *ϕ*_lim_, above which there is rapid growth of granulation tissue and no further stiffness increase takes place. So, in accordance with experimental findings [40], faster proliferation leads to a more disorganized tissue with lower stiffness properties, and vice versa.

## 4. Results

The dual-stage analysis and the constitutive model developed in the previous sections have been implemented in the in-house FE code PLCd [55]. The detailed algorithm is available in appendix A. PLCd is an implicit FE code developed in Fortran and capable of solving finite-strain nonlinear three-dimensional solid mechanics problems. It uses the direct sparse solver Pardiso [56] and a full Newton algorithm. A generic example of the E-VAC of an anastomotic cavity is presented in this section with the purpose of exemplifying the main characteristics of the model. Owing to the conceptual nature of this study, the validation example is qualitative in nature and results are verified for their plausibility.

### 4.1. Structural problem

**Figure 7.**
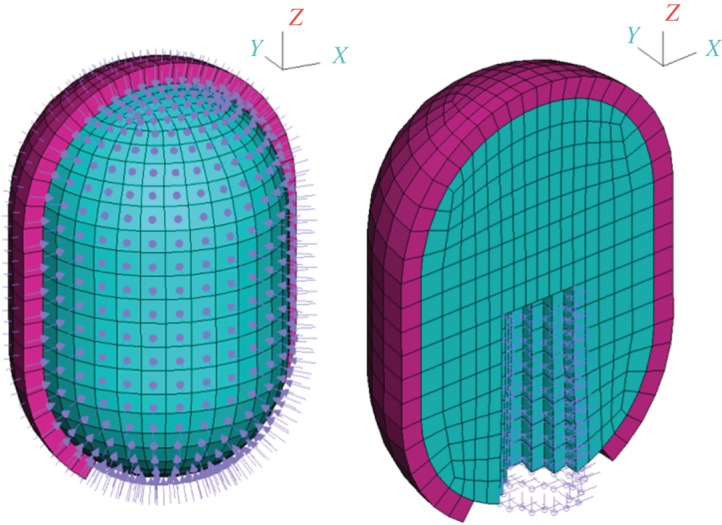
FEM model used to solve the structural problem, with 3488 elements and 4010 nodes. The foam (shown in blue) and cavity wall (shown in pink) are both meshed with linear hexahedral Q1 elements. A suction pressure is applied on the external face of the foam (left). The nodes corresponding to the area of contact between the foam and the suction tube are fully fixed (right).

The FEM model used to solve the structural problem, including the boundary conditions and loads applied, is shown in figure 7. Based on the geometry of the Eso-sponge^®^ device (B. Braun Surgical, S.A., Rubí, Spain), the foam is modelled as a cylinder with spherical tops, with a maximum length of 4.6 cm in the vertical axis and maximum diameter of 3 cm. The elements roughly corresponding to the volume where the suction tube would be inserted in the foam have been removed. The foam is glued to the tube, so the contact surface between them is fully fixed. Vertical displacements are fixed at the bottom, where the cavity ends, under the assumption that the tube is fixed during the application of vacuum. A 2.5 cm thick layer covering all the external surface of the foam, except for the bottom part where the foam would be attached to the tube, represents the cavity wall.

**Figure 8.**
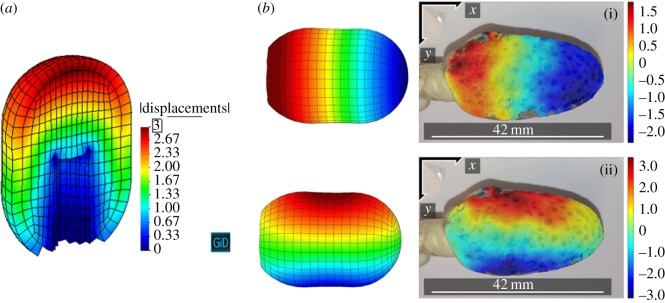
Total displacements of the FEM model of foam and cavity wall for an applied suction pressure of 125 mmHg (*a*). Comparison of the displacement field distribution between the FEM mechanical problem prediction and digital image correlation from an experimental test using a thin-walled medical latex glove (*b*). The Ncorr software [62], an open source two-dimensional digital image correlation Matlab program, was used to this aim. The Eso-sponge^®^ used in the experimental test was cut to a size that approximately matched the foam used in the FEM simulation. Experimental displacements in the *x*-axis (i, right) and in the *y*-axis (ii, right). All displacement values given in mm, real deformation plotted.

A Young’s modulus of 100 kPa and a Poisson’s ratio of 0.01 characterize the foam material. These parameters have been fitted to experimental data [57] on the compressive behaviour of water-saturated open-cell foams. Owing to the lack of experimental data on anastomotic cavity wall tissue mechanical behaviour, the parameters to model the cavity wall material are taken from experimental data of oesophageal tissue [58,59] and adjusted to produce a reasonable contraction of the wall. There is a great variability in clinical data available in the literature [31–33,45,60,61], yet it seems that to close an anastomotic defect (i.e. to reduce the cavity to less than 2 cm in depth [36]) a mean amount of between five and eight foam changes is required [34–36]. For the cavity we are modelling to close, we considered that its depth should be reduced by 2.6 cm in a maximum of eight steps, i.e. for each foam placement the depth of the cavity should shorten about 3 mm. A Young’s modulus of 144 kPa with a Poisson’s ratio of 0.3 results in a maximum displacement of 3 mm for an applied pressure of 125 mmHg on the external faces of the foam, which is the suction pressure used in E-VAC as reported in the above references. The displacements obtained from the computational analysis match quite well the experimentally obtained displacements using Eso-sponge^®^ and a medical latex glove as the cavity wall (figure 8).

### 4.2. Regeneration problem

The geometry of the FE model used to solve the regeneration problem is the cavity wall (shown in pink in figure 7) of the model for the structural problem. However, the elements used are now of type Q1P0 so that the tissue behaviour can be modelled with neo-Hookean quasi-incompressible hyperelasticity. The parameters used to characterize the tissue behaviour are given in table 1. The same initial stiffness increase index *D*_eff, 0_=0.2 is imposed on all elements of the model. The effect of varying the value of the main parameters in the regeneration problem is shown in appendix B. The displacements of the nodes in the inner face of the cavity wall obtained for the structural problem (figure 8) are imposed on the regeneration FE model to reproduce the cavity shrinkage due to 40, 80 and 125 mmHg of suction pressure. The imposed displacements are applied in one step producing a certain distribution of stresses and, then, kept fixed while the regeneration constitutive model is free to evolve triggered by said stresses. The values of the displacement imposed when the foam is replaced are the values used at the initial time (*t*=0.0 *h*), weighted by the amount the cavity was reduced during the action of the previous foams, to ensure that the suction pressure is equivalent in all foam applications.

**Table 1.** Parameters considered for the healing model in the computation of the regeneration problem. The neo-Hookean material parameter *C*_1_=*μ*/2 is obtained from the shear modulus *μ*=*E*/(2+2*ν*) using the parameter values for the cavity wall defined in the structural problem. The parameters *τ*^*d*^_0_ and *G*_*f*_ correspond to those of the linear damage model defined in [54].

parameter	value
*C*_1_	27.7 kPa
tr σ^*^_eq_	0.01 kPa
tr *σ*_1_	8.8 kPa
tr *σ*_2_	18.2 kPa
*k*_*g*_	2.5×10^8^ (kPa h)^−1^
*k*^1^_*g*_	4.5×10^8^ (kPa h)^−1^
*τ*^*d*^_0_	101.5×10^3^ kPa^0.5^
*G*_*f*_	240×10^9^ kPa mm
*D*_eff,0_	0.2
$k_{\max}^{s}$	0.001 h^−1^
*ϑ*^lim^	0.5

**Figure 9.**
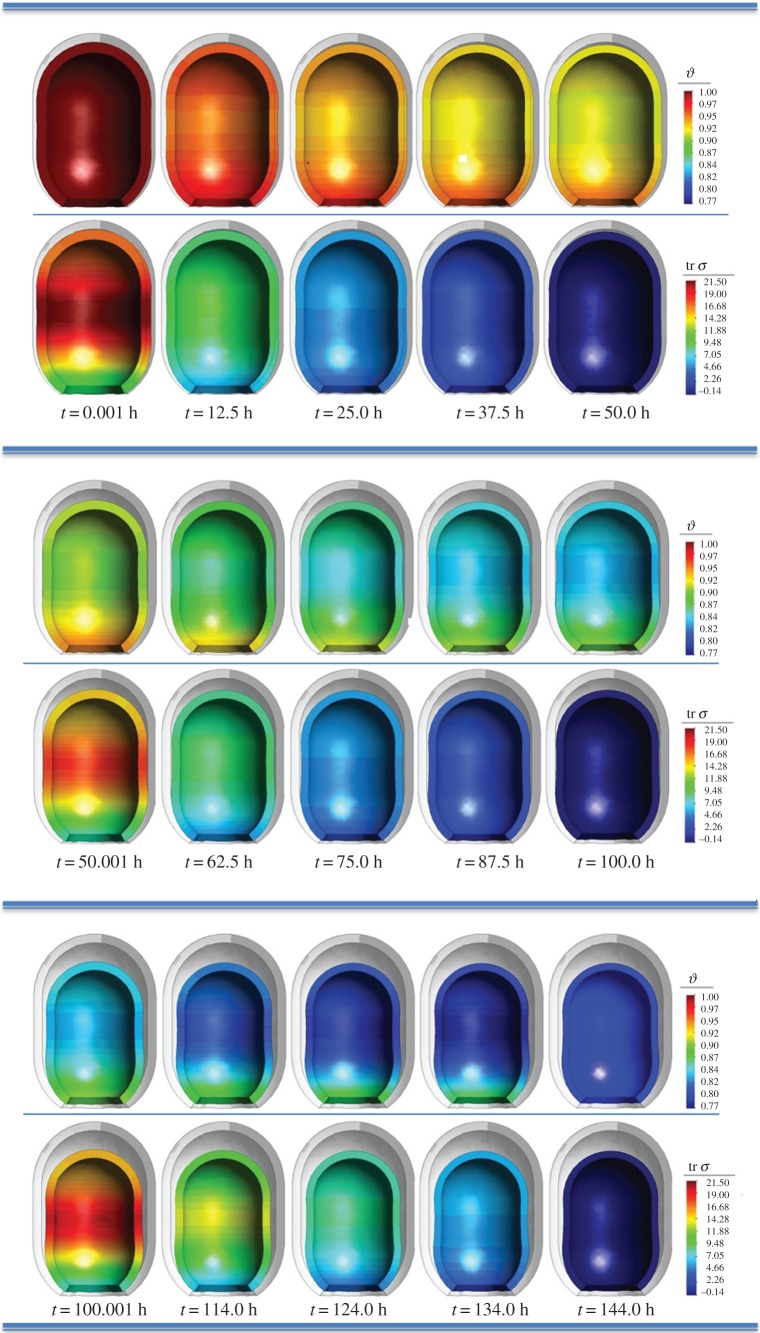
Evolution through three foam changes (at 0.0, 50.0 and 100.0 h) of the remodelling permanent stretch, *ϑ*, and the trace of the Cauchy stress tensor, tr *σ*. An imposed displacement equivalent to 125 mmHg of suction pressure is imposed for each foam change. Stresses in kPa, real deformation plotted together with original non-deformed geometry (shown in grey).

Figure 9 shows the evolution of the remodelling permanent stretch and its driving force, tr *σ*, for an imposed displacement equivalent to 125 mmHg. We observe how an increase in the remodelling permanent stretch is directly associated with a stress relaxation (decrease in tr *σ*). Once the stresses in the cavity have fully relaxed (*t*=50.0 h), a new displacement is imposed on the inner cavity of the wall. This corresponds to a foam replacement, and is associated with a sudden increase of tr *σ* and, therefore, of suction pressure. A third change of foam takes place at *t*=100.0 h.

**Figure 10.**
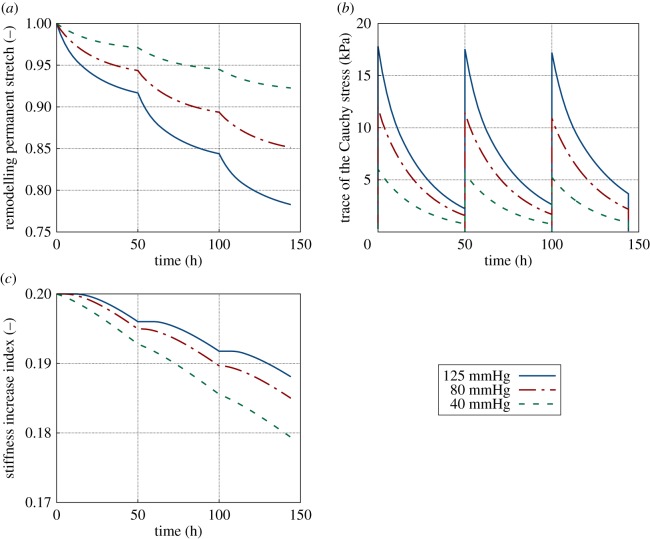
Remodelling permanent stretch, *ϑ* (*a*), trace of the Cauchy stress, tr *σ* (*b*), and stiffness increase index, *D*_eff_ (*c*), at a representative Gauss point of the inner cavity wall. Evolution through three foam changes (at 0, 50 and 100 h) considering an applied suction pressure of 125, 80 or 40 mmHg for each foam change.

The evolution of the remodelling permanent stretch, the trace of the Cauchy stress tensor and the stiffness increase index are plotted in figure 10. The sudden value increase of tr *σ* at 50.0 and 100.0 h corresponds to a foam change each, as mentioned above. Again, we clearly observe how a lower pressure results in a higher stiffness increase, but in a lower cavity reduction (lower remodelling permanent stretch).

**Figure 11.**
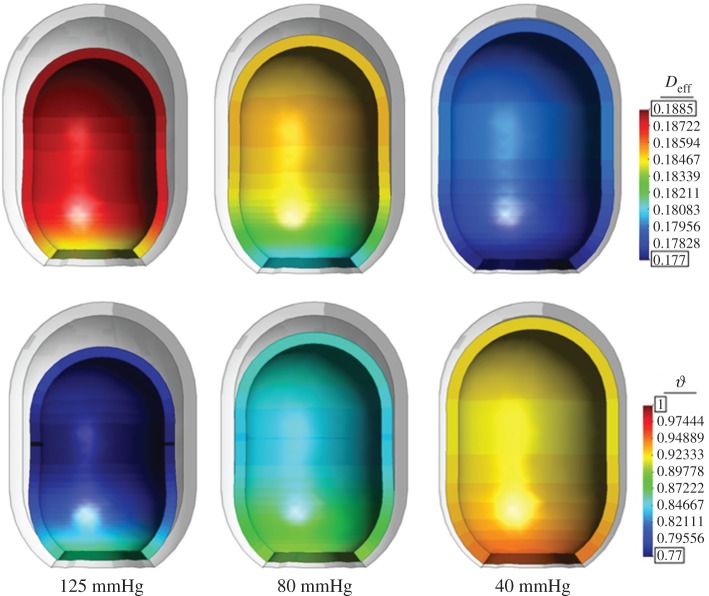
Stiffness increase index value *D*_eff_ (above), and remodelling permanent stretch *ϑ* (below) at 144.0 h (after three foam changes) for an applied suction pressure of 125, 80 and 40 mmHg. Real deformation plotted together with the original non-deformed geometry (shown in grey).

Figure 11 shows the remodelling permanent stretch *ϑ*, and the stiffness increase index value *D*_eff_ after 144.0 h of E-VAC therapy, for an imposed displacement equivalent to an applied suction pressure of 125, 80 and 40 mmHg. We observe that a higher suction pressure results in a larger reduction of the cavity (*ϑ* is lower) but in a smaller recovery of stiffness (*D*_eff_ is closer to the initial value of 0.2). This is in agreement with the notion [40] that when granulation tissue grows faster (larger reduction), it also grows in a more disorganized manner (lower stiffness, higher *D*_eff_) and shows that the coupling of the stiffness increase and the permanent deformation proposed are capable of reproducing this phenomenon.

## 5. Discussion

The computational results presented in the previous section qualitatively reproduce the E-VAC of an anastomotic leakage cavity. We are able to capture the permanent shrinkage of the cavity after each foam application and the gradual increase of stiffness of the tissue forming the cavity walls. However, the simplifications and assumptions made mean that the numerical values presented are not accurate enough to draw definitive conclusions to aid in the improvement of the technique or devices used. Nonetheless, we are confident that the computational analysis and, especially, the constitutive formulation developed here set a solid basis on which to improve, refine and adjust different aspects of the model, which we identify and discuss next.

### 5.1. Factors affecting the contraction pattern and size of the reduced cavity in the structural problem

The structural problem critically determines the final configuration of the reduced cavity. Hence, the different factors affecting the contraction pattern and size of the cavity must be correctly accounted for. For the foam, both its material behaviour and the geometrical assumptions such as shape and position of the pressure sink (i.e. where the suction tube ends) will foreseeably have an important role. With adequate experimental information, which merits further research, we could easily adapt the FEM geometrical model to better reproduce the real device, and substitute neo-Hookean hyperelasticity for a more adequate material description, including anisotropic and highly nonlinear behaviour.

Another factor to consider is the implicit assumption in our model that the internal wall of the cavity and the external surface of the foam are in permanent contact and move together when the suction pressure is applied. Combined with the application of a homogeneous pressure throughout the whole cavity wall surface, this means we cannot account for the effect of the relation between the foam size used (and whether more than one foam is inserted in the cavity at once) and the initial cavity size. Therefore, when using the same model parameters, the current model will always predict that larger cavities need more foam changes to close, regardless of the foam configuration used.

By means of experimental studies as mentioned above, we could identify how the foam size and configuration affects the distribution of the suction pressure applied on the cavity wall. The effect of using multiple foams could also be analysed, since it will probably also have an influence on the pressure distribution. This could contribute towards improving the current computational model to capture the folds observed in the simple suction experiments performed for this study (figure 2). However, these experiments show the stiffness of the cavity wall is a key factor in determining the type of structural instability generated, which underlines the need of better characterizing the structural properties of the anastomotic cavity wall.

Unfortunately, the role of the cavity wall in determining the contraction pattern and size is much harder to model with accuracy. The cavity wall layer represents the effect of both the surrounding structures and the thin layer of mucosa and fascia. Although anatomically incorrect, we could adjust the overall stiffness of the wall to reasonably reproduce the behaviour of the whole ensemble with adequate experimental data. Medical images taken immediately before and after the application of suction, either from clinical patients or using animal models, could show the change in size and shape of the cavity. Knowing the suction pressure and the properties of the foam used, we would adjust the material characterization of the cavity wall to match the experimental results using inverse parameter identification techniques.

### 5.2. Limitations of the simplifying assumptions made in modelling the regeneration problem

The focus of the regeneration problem is on capturing the permanent displacement of the inner wall, because this critically determines the closure of the anastomotic cavity. We propose permanent deformation and stiffness increase as the coupled driving mechanisms of this process, and the computational results obtained seem to confirm this hypothesis. Our focus in this initial stage has been on capturing the fundamental phenomena at play and their basic interactions, hence the selection of the simplest possible approaches in modelling continuum growth and stiffness increase.

The permanent deformation has been associated with the proliferation of granulation tissue. We used a volumetric continuum growth as an initial approach, yet tissue probably grows exclusively in the outward direction, towards the interior of the cavity. Volumetric growth allows us to represent the stress dissipation and permanent deformation of the cavity wall, even if it is not the most accurate representation of the actual reduction process. Identifying a more adequate growth model to represent the kinematics of the phenomenon in a more realistic manner is a priority we will address in future developments of the model. Through a sensitivity study of computational predictions to the growth kinematics used in the model we will be able to select a model that better fits clinical observations. However, the role of the foam design and the cavity geometry discussed in §5.1 must be studied in more depth for such a sensitivity study to produce useful insights.

The stiffness increase is based on a reinterpretation of the existing HTR constitutive model for healing in soft tissues. In the HTR formulation, the healing process models how a damaged tissue gradually recovers the stiffness of the original undamaged tissue. In the model developed here, however, the tissue we ‘recover’ is in fact a new one (scar tissue of the healed anastomotic defect), different from the initial damaged tissue (fascia and mucosa of the anastomotic cavity). We lose the original significance of the HTR formulation, but are still modelling the same effect: a stiffness increase.

The stiffness increase has been coupled to the permanent deformation owing to experimental evidence that links fast cell proliferation with a more disorganized tissue [40]. To this aim, the remodelling rate in the HTR model is now also a function of the mechanically driven growth criterion *ϕ*(*σ*^e^); see (3.9). We assume, thus, that the growth rate is explicitly driven by the suction pressure via the trace of the Cauchy stress tensor, while the rate of stiffness increase is implicitly driven by the damaged stress state of the tissue, but limited by growth. This approach produces results that are in accordance with the experimental findings [40] while introducing limited changes to the well-documented growth and healing constitutive models. Yet, we would like to further explore the relation between the two phenomena, permanent deformations and stiffness increase, as well as the choice of driving variables in these models, should specific studies on the mechanobiology of the anastomotic healing tissue become available in the future. In this sense, some questions that remain unanswered are whether growth should be limited by the stiffness increase and not the other way round; and whether the effect of the suction pressure should be explicitly accounted for in the modified HTR model.

We selected a volumetric growth and a modified HTR with the aim of minimizing the amount of parameters possible. These are phenomenological in nature and, due to the complexity of the biomechanical problem being studied and the lack of experimental data, determining their value is not a straightforward task. In fact, some of the parameter values selected in the example presented are rather arbitrary, hence we performed a short study (appendix B) to understand how modifying the value of key parameters affects the computational predictions. Our analysis reveals that changes to the mechanical criterion function illustrated in figure 5 have the highest impact on the amount of reduction in size of the anastomotic cavity at the end of the healing process.

### 5.3. Translation of computational results into clinical applications

The proposed constitutive model, phenomenological in nature, does not account for the underlying cellular processes, but can potentially reproduce the healing of an anastomotic cavity if sufficient experimental data are available to adjust the model parameters. For example, the closure of an oesophageal leakage has been reported to require several foam changes with a recommended replacement interval every 2 to 3 days [45], yet we do not have detailed information on the effect of each foam change. If we knew the foam size, suction pressure and reduction in cavity size for each foam change, we could determine a set of parameters for our computational model that reproduce the clinical observations. Once we identify several sets of parameters, for a range of geometries and conditions, we could confidently predict future modifications of the original problems used for the fitting. Then, different foam geometries and pressure values could be computationally studied and evaluated to determine optimal designs and therapeutic settings.

Once again, obtaining such data is not a straightforward task. Patient-specific medical images showing the position of the cavity before and after suction could provide information on the stiffness of the material. Comparing stiffness values in between the different foam applications would give us an idea of the stiffness increase during the healing process. Furthermore, comparing the geometry and size of the foam-free cavity between different foam changes would allow us to experimentally compute its permanent deformation. Together with a histological study of the tissue, we could further associate the mechanisms at play with the observed behaviour. In addition to confirming our assumptions on how the biological process works, this could allow including mechanistic functions into the model that take into account phenomena at the cellular level.

In this sense, a path to further explore is the effect of the foam pores in the proliferation of the wall tissue, because studies seem to indicate they might have a significant role. Microdeformation, adhesion forces and tearing of tissue with each foam replacement would have to be accounted for. Incorporating these would allow identifying optimal pressure levels and times between foam replacements to obtain a faster reduction of the cavity.

## 6. Conclusion

We have modelled the E-VAC of an oesophageal anastomotic leakage by means of a dual-stage FE analysis. We hypothesized that the closure of an anastomotic cavity could be separated into two distinct problems owing to the different time scales acting in each case. First, the *structural problem* reproduces the reduction in size of the leakage cavity due to the application of suction pressure through an open-pore polyurethane foam. Then, the *regeneration problem* reproduces the actual closure of the cavity due to tissue regeneration. In addition, two key mechanisms have been identified in the latter as drivers of the healing process: *permanent deformations* and *stiffness increase*.

We have shown through a qualitative example that the computational model is capable of reproducing the generic closure of an anastomotic cavity, confirming that the modelling assumptions agree with the clinical observations. Specifically, suction is the main driver of the healing process via the growth criterion that, based on the observed effects of VAC, allows directly associating the pressure level induced by the suction device with the main variable of this function, the mean value of the trace of the Cauchy stress tensor in the tissue.

The two mechanisms proposed to model the regeneration part of the process are consistent with experimental observations. Chiefly, when granulation tissue grows faster, it is known to be more fragile, i.e. the tissue will have lower stiffness [40]. This has been incorporated in our formulation through the dependence of stiffness increase on growth.

The main variables and parameters chosen to model the problem and their interrelations allow to reproduce the broad clinical observations. Hence, although phenomenologically, these manage to represent the fundamental characteristics of the process. However, this is a conceptual study that cannot yet inform design processes or therapy guidelines of clinical relevance. On the one hand, more experimental data are needed to improve material modelling and, on the other hand, the model assumptions need to be refined, e.g. incorporate more accurate growth kinematics and improve the interaction of growth and stiffness increase, among others. Overall, our work shows that using computational analysis to model E-VAC is feasible and can be an important tool in the future to help elucidate the mechanobiology of this understudied problem, contributing to better designs and therapy guidelines.

## Figures and Tables

**Figure 12.**
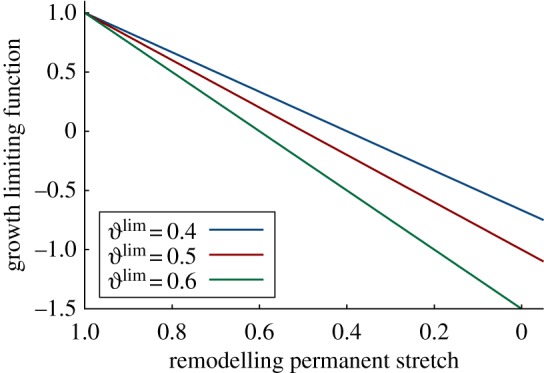
The variation of the remodelling permanent stretch limit *ϑ*^lim^ changes the slope of the growth-limiting function *f*(*ϑ*).

**Figure 13.**
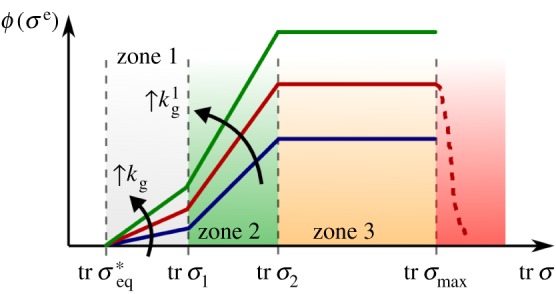
The variation of the parameters *k*_*g*_ and *k*^1^_*g*_ changes the slopes of the mechanical criterion *ϕ*(*σ*^e^).

**Figure 14.**
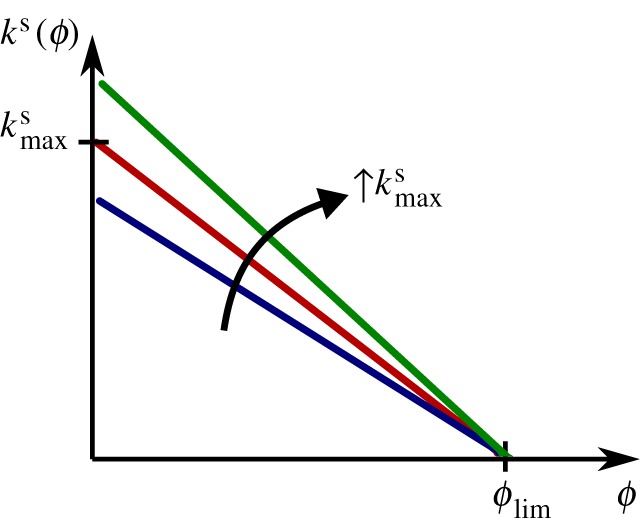
The variation of the parameter *k*^s^_max_ changes the slope of the function *k*^s^ that regulates the stiffness increase in terms of the mechanical growth rate criterion *ϕ*.

**Figure 15.**
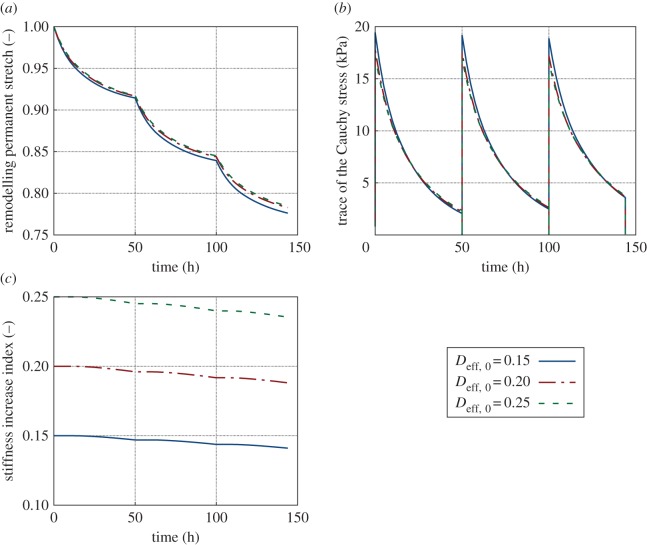
Remodelling permanent stretch, *ϑ* (*a*), trace of the Cauchy stress, tr *σ* (*b*) and stiffness increase index, *D*_eff_ (*c*), at a representative Gauss point of the inner cavity wall. Evolution through three foam changes (at 0, 50 and 100 h) considering an initial stiffness increase index *D*_eff,0_ of 0.15, 0.20 or 0.25.

**Figure 16.**
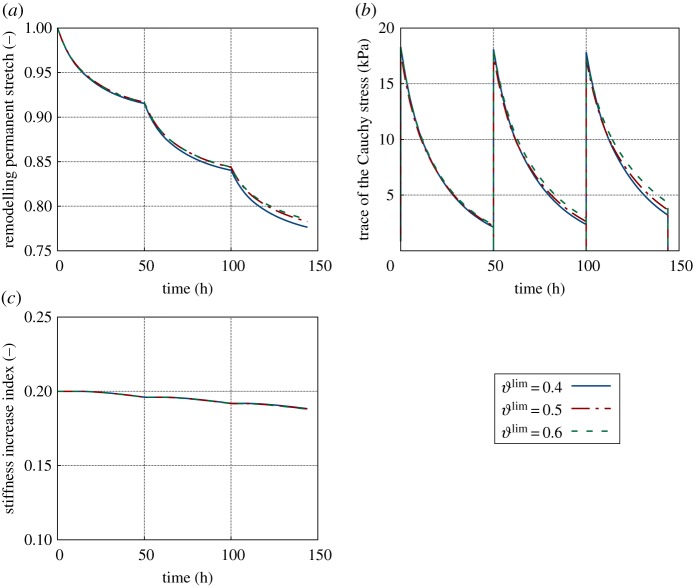
Remodelling permanent stretch, *ϑ* (*a*), trace of the Cauchy stress, tr *σ* (*b*) and stiffness increase index, *D*_eff_ (*c*), at a representative Gauss point of the inner cavity wall. Evolution through three foam changes (at 0, 50 and 100 h) considering a remodelling permanent stretch limit *ϑ*^lim^ in the growth-limiting function *f*(*ϑ*) of 0.4, 0.5 or 0.6.

**Figure 17.**
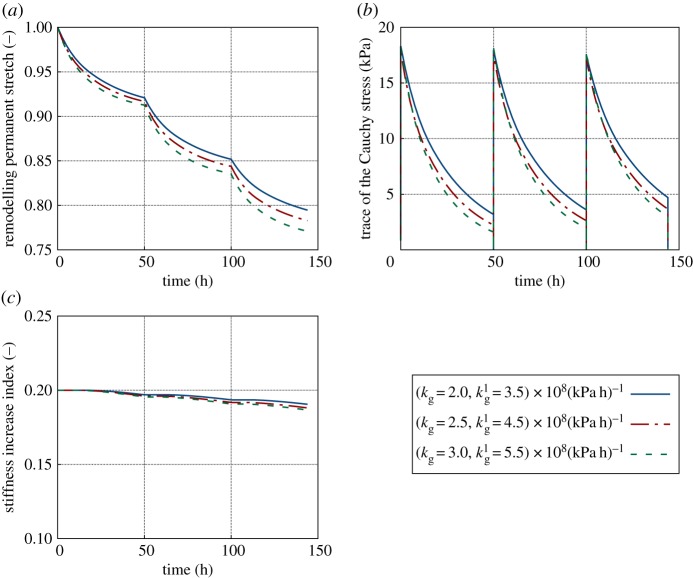
Remodelling permanent stretch, *ϑ* (*a*), trace of the Cauchy stress, tr *σ* (*b*) and stiffness increase index, *D*_eff_ (*c*), at a representative Gauss point of the inner cavity wall. Evolution through three foam changes (at 0, 50 and 100 h) considering the combined values *k*_g_ and *k*^1^_g_ in the mechanical growth function *ϕ*(*σ*^e^) of 2.0×10^8^ and 3.5×10^8^, 2.5×10^8^ and 4.5×10^8^, or 5.5×10^8^ and 3.0×10^8^ (kPa h)^−1^.

**Figure 18.**
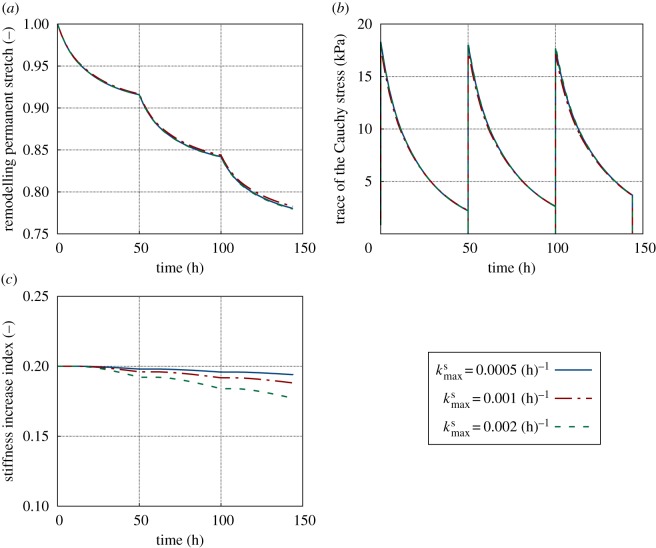
Remodelling permanent stretch, *ϑ* (*a*), trace of the Cauchy stress, tr *σ* (*b*) and stiffness increase index, *D*_eff_ (*c*), at a representative Gauss point of the inner cavity wall. Evolution through three foam changes (at 0, 50 and 100 h) considering a value $k_{\max}^{s}$ in the rate of stiffness increase *k*^s^(*ϕ*) of 0.0005, 0.001 or 0.002 (h)^−1^.

## Data accessibility

The PLCd source code is available from http://www.cimne.com/PLCd. The specific files and executable code used in this work can be found under the tab “PUBLICATIONS > JOURNAL PAPERS” by accessing the link corresponding to this publication.

## Appendix A. Numerical algorithm of the constitutive model for the regeneration problem

The numerical algorithm corresponding to the constitutive model of the healing tissue in the E-VAC of an anastomotic cavity is given in table 2. The model is implemented in a total Lagrangian framework using hybrid Q1P0 elements and a neo-Hookean hyperelastic model.

**Table 2.** Algorithm at the Gauss point level of the numerical integration in PLCd [55] of the constitutive model for healing of an anastomotic cavity.

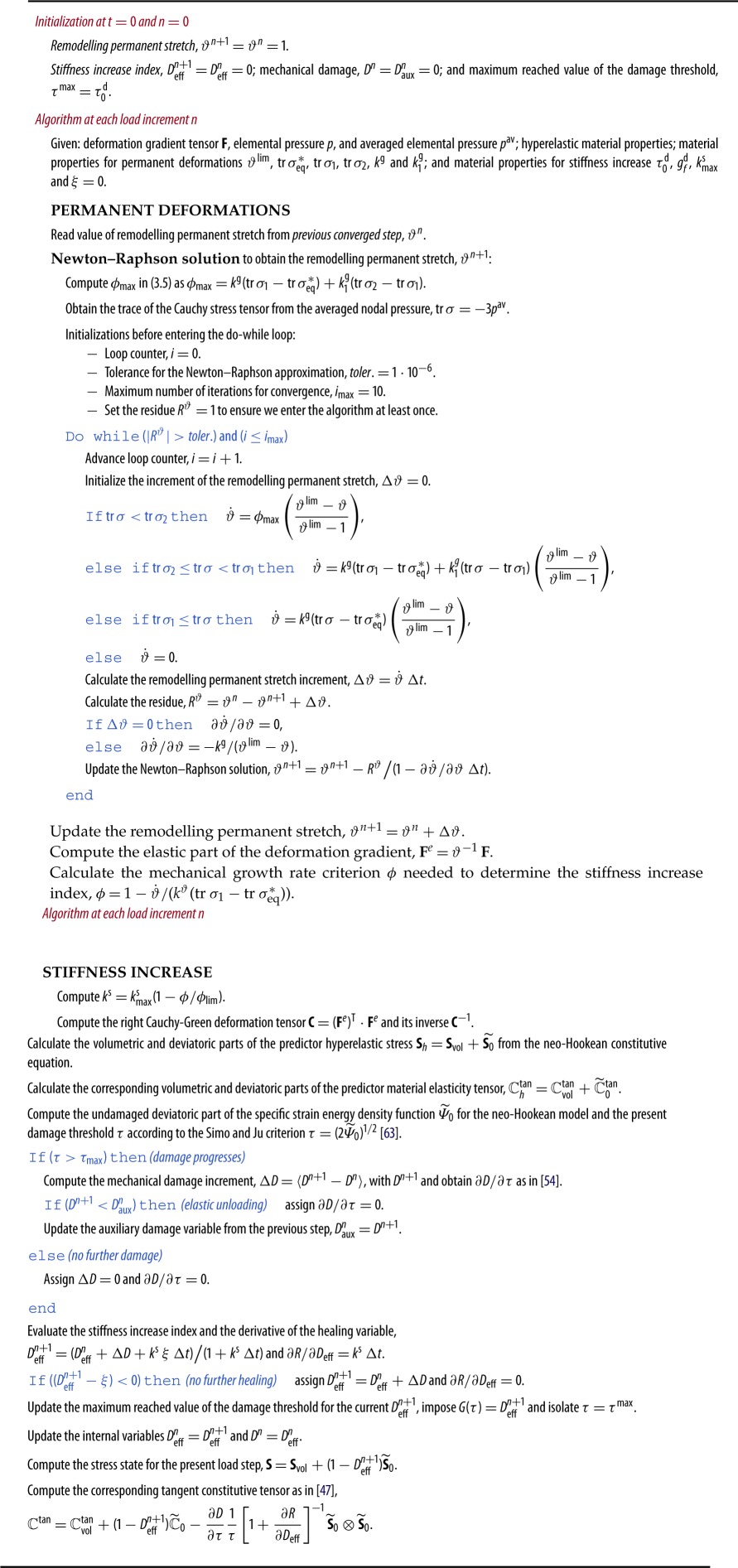

## Appendix B. Effect of varying the value of the main parameters in the regeneration problem

We study how the value of the parameters in the growth-limiting function *f*(*ϑ*) in (3.4), the mechanical criterion *ϕ*(*σ*^e^) in (3.5), and the rate of stiffness increase *k*^s^(*ϕ*) in (3.9) affect the computational solution. To this aim, we take the material parameters listed in table 1 as the values for the baseline model, and vary one at a time the material parameters of the indicated functions considering an applied suction pressure of 125 mmHg.

For the growth-limiting function, we only study the limit value of the remodelling permanent stretch *ϑ*^lim^ since *γ* simply allows to adjust the behaviour of the function from linear to polynomial. Figures 12 and 13 illustrate how the growth-limiting function changes with the different *ϑ*^lim^ values tested. In the case of the mechanical criterion, we do not modify the threshold values of the different zones shown in figure 5 because these are based on data from clinical studies. We change the slope of the mechanically driven growth criterion by simultaneously changing the value of *k*^g^ and *k*^*g*^_1_, as illustrated in figure 13. Finally, we modify the value of $k_{\max}^{s}$ in the rate of stiffness increase, which results in a change of slope of the function, as shown in figure 14. We do not look at how *ϕ*_lim_ influences the solution because changing its value is equivalent to modifying $k_{\max}^{s}$, namely, it modifies the slope of *k*^s^(*ϕ*) .

Figures 15 to 18 show how the remodelling permanent stretch, *ϑ*, the trace of the Cauchy stress, tr *σ*, and stiffness increase index, *D*_eff_ change over time at a representative Gauss point of the inner cavity wall. As can be seen in figure 15, the initial value of the stiffness increase index *D*_eff,0_ slightly affects the stress evolution and, therefore, the remodelling permanent stretch. In addition, we observe that the case with the highest *D*_eff,0_ results in the largest net increment of stiffness at the end of the process. The influence of the remodelling permanent stretch limit *ϑ*^lim^ on the results becomes more obvious as the value of the remodelling permanent stretch variable is closer to the limit value, as can be observed in figure 16. The slopes *k*_g_ and *k*^1^_g_ of the mechanical criterion are seen to directly influence how fast the recovery process is, as shown in figure 17, and are undoubtedly the parameters which influence most the final reduction of the cavity. The slope of the rate of stiffness increase *k*^s^(*ϕ*) has a direct effect on the rate of stiffness increase in the tissue, as expected, but barely modifies the remodelling permanent stretch values.
